# The dynamic behavior of lipid droplets in the pre-metastatic niche

**DOI:** 10.1038/s41419-020-03207-0

**Published:** 2020-11-17

**Authors:** Chunliang Shang, Jie Qiao, Hongyan Guo

**Affiliations:** 1 Department of Obstetrics and Gynecology, Peking University Third Hospital, 100191 Beijing, China; 2 Department of Obstetrics and Gynecology, Center for Reproductive Medicine, Peking University Third Hospital, 100191 Beijing, China; 3 National Clinical Research Center for Obstetrics and Gynecology, 100191 Beijing, China; 4 Key Laboratory of Assisted Reproduction (Peking University), Ministry of Education, 100191 Beijing, China; 5 Beijing Key Laboratory of Reproductive Endocrinology and Assisted Reproductive Technology, 100191 Beijing, China; 6 Research Units of Comprehensive Diagnosis and Treatment of Oocyte Maturation Arrest, 100191 Beijing, China

**Keywords:** Cancer metabolism, Cancer microenvironment

## Abstract

The pre-metastatic niche is a favorable microenvironment for the colonization of metastatic tumor cells in specific distant organs. Lipid droplets (LDs, also known as lipid bodies or adiposomes) have increasingly been recognized as lipid-rich, functionally dynamic organelles within tumor cells, immune cells, and other stromal cells that are linked to diverse biological functions and human diseases. Moreover, in recent years, several studies have described the indispensable role of LDs in the development of pre-metastatic niches. This review discusses current evidence related to the biogenesis, composition, and functions of LDs related to the following characteristics of the pre-metastatic niche: immunosuppression, inflammation, angiogenesis/vascular permeability, lymphangiogenesis, organotropism, reprogramming. We also address the function of LDs in mediating pre-metastatic niche formation. The potential of LDs as markers and targets for novel antimetastatic therapies will be discussed.

## Facts

- Lipid droplets have increasingly been recognized as lipid-rich, functionally dynamic organelles within tumor cells, immune cells, and other stromal cells that are linked to diverse biological functions and human diseases.
- This review discuss the current evidence related to the functions of LDs related to the following characteristics of the pre-metastatic niche: immunosuppression, inflammation, angiogenesis/vascular permeability, lymphangiogenesis, organotropism, reprogramming.
- The molecular marker of LDs, PLIN2, was significantly associated with tumor purity and infiltration of B cells, CD4+ T cells, CD8+ T cells, neutrophils, macrophages, and dendritic cells in diverse cancer types.
- We discuss the potential roles of LDs in mediating pre-metastatic niche formation.
- Treatment of the LD-associated key enzymes significantly abolished tumor cell adhesion to endothelial cells and reduced the recruitment of immune cells in pre-metastatic niche.

## Open questions

- What is the definition of ‘LDs’ and its biogenesis in tumor microenvironment cells?
- What is the maturation process of LDs in tumor pre-metastatic niche?
- How does LDs regulate pre-metastatic niche formation?
- LDs reprograming represents promising cancer therapy, what are the limitations and adverse reactions? How to improve treatment efficiency?
- What should be further studied about LDs in pre-metastatic niche?

## Introduction

Metastasis is the primary cause of cancer morbidity and mortality^1^. Tumor metastasis is a complicated process termed the invasion-metastasis cascade, which involves multiple sequential and interrelated steps and several biochemical events. The metastatic cascade comprises the following sequential steps^2^: (1) tumor cells gain invasive activity and detach from the primary tumor site; (2) disseminated tumor cells (DTCs) break through the basement membrane (BM) and extracellular matrix (ECM) and invade the blood and/or lymphatic vessels; (3) circulating tumor cells (CTCs) escape immune elimination and survival in lymphatic and/or vascular circulation; (4) CTCs initiate extravasation, crossing from the vascular lumen into the organ parenchyma; and (5) CTCs survive and subsequently proliferate to form overt metastases in a secondary organ/site. Therefore, metastatic dissemination is not only determined by the mechanical dynamics of hematogenous flow^3^; Steven Paget’s “seed and soil” theory is also a pivotal milestone in explaining metastatic colonization^4,5^. However, why are only a few tumor cells capable of becoming DTCs and leaving the primary tumor? Are DTCs more prone to lymphatic or blood metastasis? Why does the development of metastases occur selectively in certain organs but not others^6,7^? Moreover, what is the interplay between intrinsic tumor cell properties and the interactions between tumor cells and multiple microenvironments?

A wealth of research has revealed that distant metastatic organs are not passive recipients of CTCs but instead selectively and actively prepare the secondary microenvironment in response to the primary tumor for CTC colonization before the arrival of CTCs^8,9^. These preconditioned microenvironments are termed pre-metastatic niches (PMNs) and were first described by Kaplan, R. N. et al.^10^. The significance of the PMN has received increasing attention in recent years^11,12^. Cao^8^ summarized six characteristics that define the PMN: inflammation, immunosuppression, angiogenesis/vascular permeability, lymphangiogenesis, organotropism, and reprogramming. Furthermore, primary tumor-derived molecular components (soluble factors and extracellular vesicles^13^), tumor-recruited bone marrow-derived cells (BMDCs)^14^, regulatory/suppressive immune cells^15^ and the future metastatic organ-specific microenvironment have been identified as the crucial determinants for PMN formation. Based on the above characteristics and PMN components, the formation of PMNs covers almost all of the key events of tumor metastasis. It is extremely important to explore the spatiotemporal regulatory relationship between these PMN characteristics of and who the probable prime culprit is at different stages of PMN formation.

**Fig. 1:**
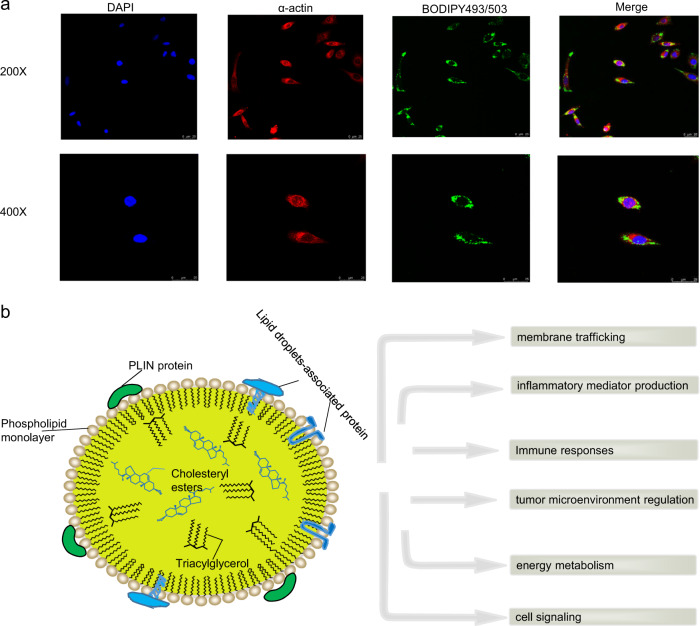
Structure and basic function of lipid bodies. **a** Fluorescence microscopy image shows LDs with double staining with BODIPY 493/503 dye (green) and Hoechst in the macrophage cells. **b** Schematic representation of the structural composition of LD and the basic function of LD is listed in the right.

Lipid droplets are cytoplasmic lipid-rich organelles that are recognized as platforms with multiple functions in cancers^16,17^. LDs were initially perceived as static energy storage deposits, such as glycogen particles but are increasingly described as dynamic and inducible spherical organelles that exist in almost all eukaryotic cells (Fig. 1a). Unlike most other intracellular organelles, LDs are surrounded by a monolayer of phospholipids composed of a hydrophobic core of triacylglycerol (TAG) and cholesteryl esters (CEs) and coated with hundreds of proteins, including the perilipin (PLIN) family^18^ (Fig. 1b). Among the PLIN family, PLIN2 as a LD marker, is universally expressed in nearly all tissues^19^. In the early 1960s, LDs were reported to be abundant in tumor cells and might assist in the diagnosis of Burkitt’s tumor^20,21^. With key research findings showing that prostaglandin E2 (PGE2) synthesis in LDs can promote colon cancer cell proliferation^22^, considerable attention has been paid to the role of LDs in cancer involving membrane trafficking, energy metabolism, cell signaling, immune responses, inflammatory mediator production, and tumor microenvironment regulation^23–25^. Interestingly, growing evidence indicates that, compared with the Warburg effect in cancer cells, reprogramming lipid metabolism may provide a selective advantage for the tumor metastatic process^26^. Our study^27^ also confirmed that the accumulation of LDs enhances lymphangiogenesis and the epithelial–mesenchymal transition (EMT) of tumor cells, resulting in lymph node (LN) metastasis of cervical cancer. Based on this information, LDs may be the prime culprit participating in each step of PMN formation.

We aimed to summarize the functions of LDs according to the characteristics of PMN formation and finally provide new strategies in the control of cancer metastasis by targeting LD biogenesis.

## Lipid droplet biogenesis

**Fig. 2:**
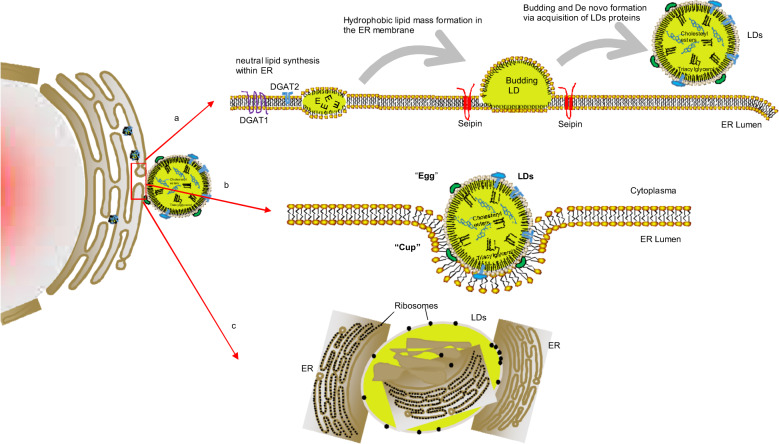
Models of lipid droplet formation. **a** The “budding model”: The neutral lipids are sequestered to form hydrophobic lipid mass between the two leaflets of the phospholipid bilayer of the ER. Once the nascent lipid bodies reach a certain size, they leave the endoplasmic reticulum by budding, and then the phospholipid bilayer encased on the droplet surface is rearranged to form a phospholipid monolayer. **b** The “egg cup model”: The lipid droplet occurs preferentially closely apposed the ER membrane outside the ER, just like an egg cup (the ER) holding an egg (the lipid droplet). **c** The “enfolding model”: The presence of ribosomes, ER-like membranes, membrane-associated and transmembrane spanning proteins within lipid droplets cores.

Despite major advances in recent years, the mechanism of LD formation is still poorly understood. LDs can either form de novo or from preexisting LDs by fission^28^. In eukaryotes, the neutral lipid core in the LD is synthesized in the endoplasmic reticulum (ER)^29^. The prevailing models classify LDs as ER-derived organelles that likely form de novo in eukaryotes^30^. There are three hypotheses to explain LD formation and their interaction with the ER. First, the budding model is still largely accepted^16,31,32^. The enzymes involved in lipid metabolism are responsible for neutral lipid synthesis and accumulate in distinct domains of the ER. These neutral lipids are sequestered to form hydrophobic lipid masses between the two leaflets of the ER phospholipid bilayer. Once the nascent lipid bodies reach a certain size, they leave the ER by budding, and then the phospholipid bilayer encased on the droplet surface is rearranged to form a phospholipid monolayer (Fig. 2a). The second model is called the egg cup model and was proposed by Robenek and colleagues based on freeze-fracture electron microscopy analysis^33^. This hypothesis suggests that the LD is not localized between the leaflets of the ER membrane; rather, it occurs preferentially closely appose to the outside of the ER membrane, similar to an egg cup (the ER) holding an egg (the LD). Adipophilin clusters in the cytoplasmic leaflet of the ER transfer lipids from the ER to the nascent lipid body surface (Fig. 2b). The third model is the enfolding model and explains that LDs are formed by incorporating multiple loops of ER membranes. The presence of ribosomes, ER-like membranes, and membrane-associated and transmembrane spanning proteins within LD cores provides strong evidence to support this hypothesis^34,35^ (Fig. 2c).

Although LDs are ubiquitous organelles, the size of LDs varies widely between cells or even within the same cell, ranging from a few dozen nanometers to hundreds of micrometers^36,37^. These prominent differences often reflect the metabolic states of cells and have been linked to cancer progression^38,39^. LD fusion is one of the most widely studied mechanisms for controlling droplet size, which leads to larger droplet formation by merging multiple droplets^31^. Cell death-inducing DFF45-like effector (CIDE) family proteins, especially Cidea and Fsp27, have emerged as key regulators of LD size^37^. CIDE proteins are extensively riveted on the LD surface by carboxy­terminal amphipathic helices and form dimers. Until they encounter another CIDE-positive LD, these dimers from different LD surfaces form stable transorganelle oligomers to achieve LD fusion^40,41^. Fsp27 enrichment at LD contact sites recruits Rab8a-GDP and AS160 to form a ternary complex to promote droplet fusion, thus inducing lipid transfer from the smaller (donor) to the larger (acceptor) LD^42^. Otherwise, phosphatidylcholine (PC) acts as a surfactant to stabilize LDs and prevent their coalescence, which generated by cytidylyltransferase (CCT) activation on surfaces of expanding LDs levels through the Kennedy pathway^43^.

## Proinflammatory effect of lipid droplets in the pre-metastatic niche

The inflammatory reaction is an important driver of cancer development and metastasis. The establishment of an inflammatory microenvironment contributes to the seeding, survival, and proliferation of CTCs in the PMN. LDs are well known for their unique capacity to act as specialized hubs of inflammatory mediators in leukocytes (e.g., macrophages, neutrophils, and eosinophils)^35,44,45^. As early as the 1980s, the studies showed that normal human neutrophils contained few LDs and the morphology of LDs in these neutrophils was similar to that of LDs in eosinophils, mast cells, and macrophages^42,43^. In contrast, LD numbers increased in neutrophils involved in host responses to tumors or in inflammatory reactions in biopsied tissues^42^. Except for the difference in LD size in normal neutrophils and in inflammatory responses, the LDs were morphologically similar^42^. Macrophages, as the most abundant leukocytes in solid tumors, are a double-edged sword in the tumor microenvironment. M1 macrophages promote the inflammatory response, while M2 macrophages promote the opposite response. In non-PMN inflammatory states, the infection alone induced a signifcant increase in the numbers of light dense LDs compared to non-infected controls, in which LDs preferentially acts as electron dense organelle. And these results verified that LDs may act as structural markers of the innate immune response in phagocytic cells^46^. In PMN states, inhibition of LD formation by C75 shifts M2-polarized macrophages to M1 polarization, thus helping to shape the inflammatory microenvironment, which may ultimately facilitate tumor rejection^47^.

Eicosanoids, including prostaglandins and leukotrienes, are a family of arachidonic acid (AA)-derived signaling lipids that have important roles in inflammation and cancer^48–50^. Lipid bodies are particularly active sites for the metabolism of arachidonic lipids in leukocytes. Cytosolic phospholipase A2 (cPLA2), the rate-limiting enzyme in the formation of eicosanoids, and its activating protein kinases extracellular signal-regulated kinase 1 (ERK1) and ERK2 colocalize at LDs, thus providing strong evidence for the major role for LDs in eicosanoid synthesis^51^. Neutrophils, as the main driver in establishing the PMN, secrete leukotrienes to aid lung colonization by selectively expanding the subpool of highly tumorigenic breast cancer cells^52^. Proinflammatory PGE2 is the most physiologically abundant prostaglandin and has a predominant role in promoting tumor metastasis^49,53^. PGE2 is induced by primary tumor-derived vascular endothelial growth factor (VEGF) and functions as a chemoattractant to recruit BMDCs to distant organs for forming discrete, fertile fields of pre-metastatic “soil”^54^. cPLA2α inhibition has long been suggested as a promising anti-inflammatory strategy^55^. The selective cPLA2α inhibitor CIX specifically impedes the migration of metastatic cells by interfering with the MyD88-dependent Toll-like receptor (TLR) and nuclear factor kappa B (NF-κB) inflammatory signaling pathways and reducing PGE2 production^56^. Cyclooxygenase (COX), the key enzyme involved in eicosanoid biosynthesis, is specifically localized to LDs in activated inflammatory cells^57^. COX-2 has been identified as a key metastasis progression gene and facilitates the breaching of lung capillaries by CTCs to seed pulmonary metastases^58^.

Proteins involved in AA transport have also been shown to localize within LDs. S100A8/A9 is the major arachidonate carrier that was identified by proteomic analysis of neutrophil lipid bodies and translocates unsaturated fatty acids (FAs) from the cytosol to the membrane^35,59^. S100A8/A9 induces the expression of serum amyloid A (SAA) 3 by stimulating NF-κB signaling in a TLR4-dependent manner and thus recruits Mac1+ myeloid cells to secondary metastatic lung sites to create an inflammatory state. This inflammation-like state promotes the migration of primary tumor cells to lung tissues and enhances PMN formation^60^. Another study verified that tumor necrosis factor alpha (TNFα), originally produced by primary tumor cells, activates the S100A8–SAA3–TLR4 cascade in the metastatic lung in a paracrine manner, thus stimulating Clara cells to express SAA3 to maintain an inflammatory state in the pre-metastatic lung niche^61^.

## Lipid droplets induce immune suppression or immune surveillance in the pre-metastatic niche

**Fig. 3:**
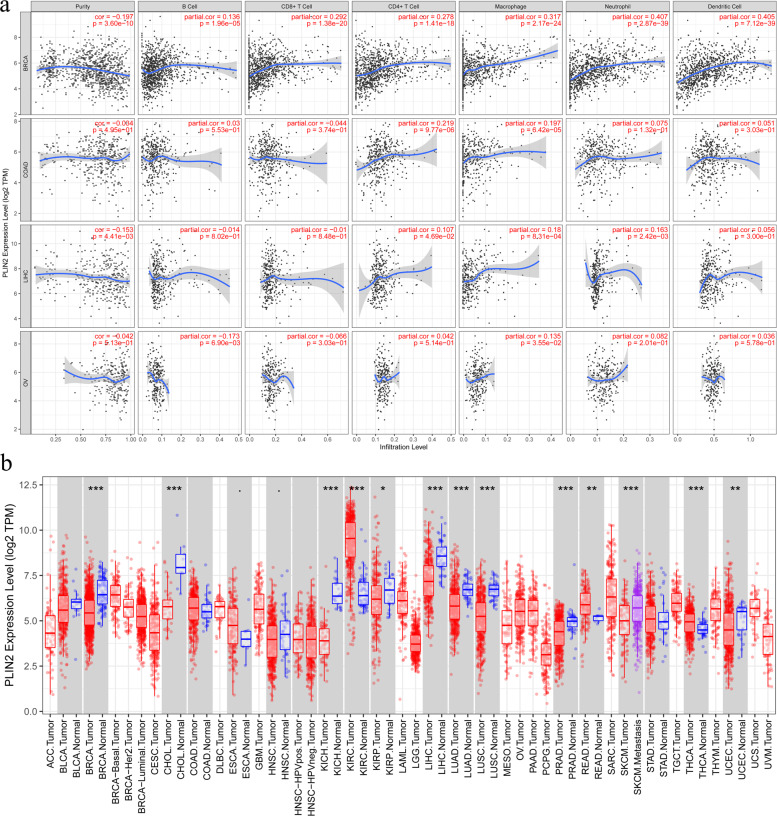
Association of PLIN2 expression with immune infiltration in cancers. **a** The correlation of PLIN2 expression with immune infiltration level in diverse cancer types. **b** The differential expression between tumor and adjacent normal tissues for PLIN2 across all TCGA tumors.

The escape of tumor cells from immune surveillance is a key feature for distant metastasis. Compelling evidence indicates that myeloid-derived suppressor cells (MDSCs), dendritic cells (DCs), and tumor-associated macrophages (TAMs) are key immunosuppressive cells that promote the establishment of PMNs^62,63^. Recent studies indicate that LDs, as sites of eicosanoid production in the previously mentioned immunosuppressive cells, modulate the crosstalk between tumors, and these cells^17,64–66^. We utilized TIMER to explore potential associations between the expression of PLIN2 and both tumor purity and infiltration of immune cells in various cancers. PLIN2 was significantly associated with tumor purity and infiltration of B cells, CD4+ T cells, CD8+ T cells, neutrophils, macrophages, and DCs (Fig. 3a). Interestingly, the role of LDs may be completely reversed in different cancers (Fig. 3b). The lowering LD abundance contributes to the migration and metastasis of triple-negative breast cancer through stimulation of fatty acid oxidation to fuel OxPhos^67^. However, the low expression of PLIN2 was associated with favorable prognosis for the stage I–III breast cancer patients^68^. This counterintuitive phenomenon for breast cancer may due to the inconsistent tumor pathological staging and biological behavior in these studies. In colorectal cancer, LD accumulation drives cell-death resistance to 5-fluorouracil and oxaliplatin treatments and is associated with a reduction in CD8+ T cell infiltration in metastatic tumors of CRC patients^69^. These data further provide the evidence that LDs could provide an inflammatory environment leading to immunosuppressive mechanisms^70^.

MDSCs are a heterogeneous population of immature myeloid cells that regulate PMN formation and development^14^. Recent studies have shown that tumor-derived cytokines (granulocyte colony-stimulating factor (G-CSF) and granulocyte-macrophage colony-stimulating factor (GM-CSF)) induce the upregulation of lipid transport receptors in tumor-infiltrating MDSCs, which triggers lipid influx, LD biogenesis, and oxidative metabolism. Furthermore, the inhibition of LD formation by DGAT blocks the immunosuppressive function of MDSCs^71–73^. Using antibody-based single-photon emission computed tomography (SPECT) imaging, Eisenblaetter, M et al. established that S100A8/A9 released by MDSCs are a surrogate marker for immunomodulation in the context of pre-metastatic lung tissue priming^74^. For the non-PMN inflammatory states, MDSC suppressive activity was also triggered by lactoferrin and mediated by PGE2, and S100A9/A8 proteins^75^. It provides another piece of evidence for the role of inflammation in PMN. In addition, tumor cells can induce programmed death-ligand 1 (PD-L1) expression in MDSCs by affecting PGE2 metabolism, thus stimulating immune suppression and alleviating the antitumor immune response in the tumor microenvironment^76^.

DCs are professional antigen-presenting cells that specialize in presenting captured tumor-associated antigens to cytotoxic CD8+ T cells via major histocompatibility I (MHC-I) complexes^77^. Previous studies identified that complementation of Igtp-deficient DCs by retroviral infection restored both LDs and efficient cross-presentation, which suggests that LDs have a direct effect on cross-presentation in non-PMN inflammatory states^78^. DCs cells are found within the PMN; however, after splenectomy, which blocks the source of DCs, the number of DCs within the lung tissue surrounding metastases is markedly reduced^79^. Studies have demonstrated that the accumulation of LDs in DCs is not only a consistent phenomenon found in tumor-draining and distant LNs, but these cells also have a profound defect in the ability to present antigens in cancer^80,81^. There are conflicting data showing that saponin-based adjuvants (SBAs), a cancer vaccine component, uniquely induce intracellular LD formation in parallel with cross-presentation in CD11b+ DCs in vitro and in vivo^64^. These differences may be due to the quality of LDs rather than the quantity and are related to dysfunctional DC antigen presentation^17,82^.

TAMs are the major tumor-infiltrating leukocytes and support persistent cancer cell growth at metastatic sites by preventing immune destruction. The novel lipid mediator CYP4A/20-HETE in TAMs promotes lung PMN formation through the M2 macrophage-derived factors transforming growth factor beta (TGF-β), stromal-derived factor 1 (SDF-1), and VEGF via signal transducer and activator of transcription 3 (STAT3) signaling^83^. Wu et al. provided evidence that the immunosuppressive phenotype of TAMs is controlled by LD-dependent FA metabolism that depends on the mammalian target of rapamycin (mTOR)-signaling pathway^84^. Additionally, epidermal FA-binding protein (E-FABP)-expressing TAMs produce high levels of interferon beta (IFNβ) through upregulation of LD formation and further enhance recruitment of NK cells in response to antitumor activity^85^. In contrast, caspase-1 inactivates medium-chain acyl-CoA dehydrogenase (MCAD) by cleaving peroxisome proliferator-activated receptor gamma (PPARγ) and thus inhibits FA oxidation, leading to the accumulation of LDs in TAMs. This stimulates TAM differentiation toward a protumorigenic phenotype^86^. Therefore, further work to elucidate the functions of TAM LDs in PMN formation is necessary.

## Lipid droplets increase angiogenesis and vascular permeability in the pre-metastatic niche

**Fig. 4:**
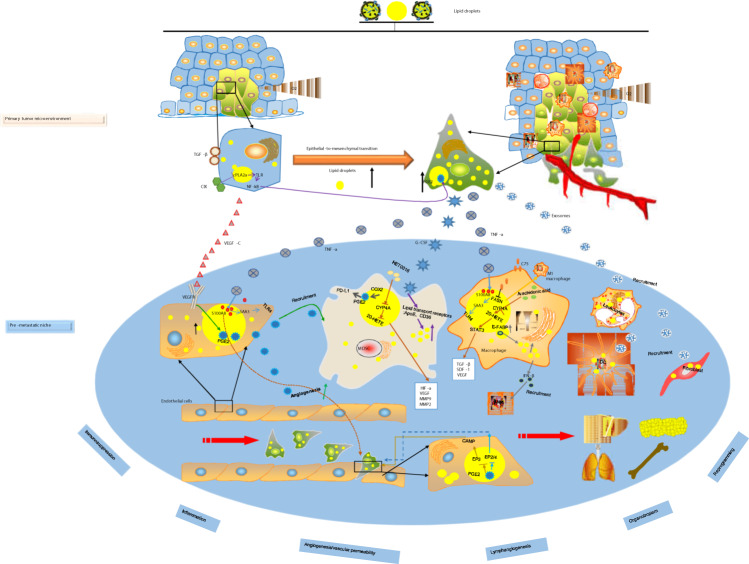
Lipid droplets roles in pre-metastatic niche. LDs promote the upregulation of inflammatory molecules, induce immune suppression, increase angiogenesis and vascular permeability, promote lymphangiogenesis, determine organotropism metastasis and promote matrix remodeling.

The PMN may increase metastatic homing by inducing angiogenesis and vascular permeability. The role of LDs in PMN characteristics are still not fully established (Fig. 4). Studies have reported that primary tumor-derived VEGF induces PGE2 expression and thus increases angiogenesis in pre-metastatic lungs^54^. In addition, PGE2 may also regulate the release of VEGF by mouse pulmonary endothelial cells via the EP2 receptor pathway and thereby promote pulmonary angiogenesis in secondary metastatic organs in a breast cancer mouse model^87^. In experimental melanomas, the FA synthase inhibitor orlistat significantly inhibits lung colonies by reducing both metastases and peritumoral angiogenesis^88^. It is worth noting that LDs may lay a spatial foundation for vasculogenic mimicry by a spatial placeholder in the tumor microenvironment^89–91^. In contrast to the endothelium-dependent blood vessels formed by the proliferation and migration of endothelial cells, vasculogenic mimicry is formed by tumor cells. Targeting the AA pathway by inhibiting the synthesis of 20-HETE results in decreased angiogenesis and vascular mimicry in the metastatic niches^92^. The definite function of vasculogenic mimicry formation mediated by LDs needs to be further elucidated in the PMN. Similarly, increased vascular permeability is an initial step in PMN formation and facilitates lung metastasis by promoting the extravasation of tumor cells^93^. Activation of the PGE2-EP3 receptor signal induces a cAMP-dependent enhancement of the endothelial barrier, resulting in hypopermeability. In contrast to EP3 receptor agonists, EP2 and EP4 receptor agonists induce vasodilatation and lead to hyperpermeability^94^. Research has also found that TGFβ-induced vascular hyperpermeability may facilitate extravasation of CTCs by modulating the expression of S100A/9 during the pre-metastatic phase^95^. Unfortunately, there is no direct experimental or clinical evidence to elucidate the role of LDs in vascular permeability in the PMN.

## Lipid droplets may promote lymphangiogenesis in the pre-metastatic niche

Lymphangiogenesis in the PMN is a potent inducer of tumor metastasis to distant LNs and distant organs. The lymphatic vasculature is lined by lymphatic endothelial cells (LECs) and is required for tissue fluid homeostasis, lipid absorption, and immune surveillance. LEC-specific loss of carnitine palmitoyltransferase 1A (CPT1A), a rate-limiting enzyme in FA β-oxidation, increases acetyl coenzyme A production by the transcription factor PROX1 and thus promotes lymphangiogenesis^96^. Consistently, FA synthase (FASN) inhibition with orlistat reduces lymphangiogenesis, leading to a decrease in the number of LN metastases in a melanoma model^97^. Our previous study^27^ also illuminated that LNMICC (lncRNA associated with LN metastasis in cervical cancer) promoted lymphangiogenesis in the LN PMN by recruiting the nuclear factor nucleophosmin (NPM1) to the FA-binding protein FABP5. A recent study reported that COX-2-derived PGE2-EP3 signaling enhances lymphangiogenesis by facilitating DC recruitment in regional metastatic LNs before the arrival of Lewis lung carcinoma cells in a murine model^98–100^.

It is well known that excessive LD accumulation in adipocyte cells leads to several human diseases^101^. LDs are increased in tumors and M2 macrophages in high-fat diet (HFD) mice^102^. HFD increases M2 macrophages in adipose tissues, and adipocytes stimulate VEGF-D expression in M2 macrophages. This crosstalk between adipocytes and M2 macrophages increases lymphangiogenesis in the tumor microenvironment. In addition, adipocytes increase C–C motif chemokine ligand 19 (CCL19) and CCL21 expression in LECs and C–C chemokine receptor type 7 (CCR7) expression in tumor cells, which attracts more tumor cells to the lymphatic vessels. Therefore, LDs may play a pivotal role in the LN PMN by participating in lymphangiogenesis (Fig. 4).

## Lipid droplet functions in metastatic organotropism

Different cancer types and subtypes display distinct organotropism, and metastatic organotropism may be innately related to the PMN^103^. Authoritative research^104^ confirms that cells expressing high levels of the FA receptor CD36 and lipid metabolism genes are unique in their ability to initiate metastasis. Similarly, that same study also found that palmitic acid or an HFD specifically enhances the metastatic potential of CD36-dependent metastasis-initiating cells. This may be an intrinsic property of tumor metastatic organotropism (Fig. 4).

LD-related regulatory molecules also participate in the process of metastatic organotropism. Primary breast cancer cell-derived VEGF induces PGE2 production in the lung PMN, which results in preferential homing of tumor cells to the lung^54^. Moreover, cancers have a high predisposition to metastasize to the omentum and bone marrow, which is affected by the presence of adipocytes that are rich in LDs. The omentum, a visceral white adipose tissue depot, induces the expression of CD36 and is associated with increased lipid accumulation^105^. Approximately 80% of all serous ovarian carcinomas metastasize to the omentum, and studies have demonstrated that adipocytes enhance angiogenesis and drive the establishment of an immune microenvironment in the omental PMN^106^. In bone, a depot for marrow adipose tissue is also a site of significant metastatic growth. Hematopoietic progenitor cells (HPCs) expressing VEGFR1 reside within specified niches of the bone marrow, home to pre-metastatic sites and form cellular clusters to attract CTCs^10^. Therefore, the selectively enriched LDs in different future metastasis organs may endow cancer metastasis with organotropic characteristics.

## Lipid droplet-dependent stromal reprogramming involvement in the pre-metastatic niche

Stromal reprogramming acts as “soil” and takes place at secondary metastatic sites to form microenvironments that support the outgrowth of metastases. The stromal environment of the PMN is mainly composed of cancer-associated fibroblasts (CAFs), endothelial cells, and ECM^11^. LDs have properties that affect both cancer cells and stromal progression in the PMN (Fig. 4). Pancreatic CAFs, which are pancreatic stellate cells, are the resident lipid-storing cells of the pancreas and have been proposed as one of the major drivers for metastatic niche formation^107,108^. Quiescent pancreatic stellate cells are activated by FAs, resulting in an inflammatory environment. This FA-mediated stromal reprogramming of pancreatic stellate cells induces inflammation and fibrosis in the pancreatic cancer environment^109^. HFD-induced fatty liver activates hepatic stellate cells and promotes ECM deposition, which creates a permissive prometastatic microenvironment for hepatocellular carcinoma^110^. Moreover, endothelial cells secrete the proinflammatory factors S100A8 and S100A9 in pre-metastatic lungs to facilitate distant metastasis^13,60^. In addition, significantly increased lipid levels and LDs were observed in cancer cells under hypoxia^111^. Further research found that exosomes secreted by cancer cells under hypoxic conditions promote matrix metalloproteinase (MMP) activity in several putative metastatic sites^112^. However, a large number of studies have confirmed that BMDCs promote matrix remodeling in the PMN and elevate fibronectin (FN) expression. The deposition of FN in hepatic stellate cells promotes the stagnation of myeloid-derived macrophages and neutrophils in the liver, completing the formation of the PMN^10,113^. However, how the LDs contained in these stromal cells regulate stromal remodeling in the PMN has not been elucidated in detail.

## Dynamic changes in lipid droplets occur throughout pre-metastatic niche formation

The presence of a PMN suggests that the metastatic process may be an ordered series of pathological events (Fig. 4). Cao^8^ summarized PMN formation into the following four stages: priming, licensing, initiation, and progression. Because LDs are functional organelles that are found in almost all cells, studying the role of LDs in PMN formation is more meaningful.

### The priming phase

Chronic acidosis, such as hypoxia, induces LD formation in cancer cells in the primary tumor microenvironment, with TGF-β2 as a key signaling actor to drive LD biogenesis through the uptake of FAs. Furthermore, TGF-β2-stimulated LD accumulation promotes acidosis-adapted cancer cell invasiveness by inducing EMT, which represents a prerequisite to support distant metastatic spreading^114,115^. In addition, primary tumor cells produce exosomes that deliver various kinds of functional molecules, including metabolites (lipids and regulatory proteins), contribute to MDSC recruitment in secondary target organs and are taken up by organ-specific cells to prepare the PMN^116^. The role of LDs in exosomes as communication messengers needs to be further explored.

### The licensing phase

BMDCs and other stromal cells are recruited to the early PMN, and the ECM is actively reprogrammed to support the organ-specific microenvironment, which prepares for potential seeding and colonization of CTCs. We have summarized the function of LDs in this process in the previous sections.

### The initiation phase

BMDCs and other immune cells secrete chemokines to attract CTCs to niche sites. For example, cancer cells metastasize to other organs by upregulating the AA-induced surface chemokine CXCR4; thus, CXCR4 on the CTC surface binds to its ligand CXCL12/SDF-1, which is expressed in the early PMN^117^. Similarly, LECs express a high level of the CCR7 ligands CCL19 and CCL21, and COX-2-induced upregulation of CCR7 in cancer cells promotes the migration of cancer cells toward LECs, enhancing lymphatic invasion^118^. Moreover, exosomes from the bone marrow promote cancer cell dormancy, and some CTCs enter dormancy until the niche environment becomes suitable. The mechanism by which LDs regulate the dormancy of CTCs remains unclear.

### The progression phase

Metastatic tumor cells escape dormancy, proliferate, and progress at the mature metastatic niche, leading to macrometastases. Epoxyeicosatrienoic acids (EETs), the cytochrome P450 epoxygenase metabolite of AA, stimulate extensive multiorgan metastasis and escape from tumor dormancy at the site of metastasis in a variety of mouse cancer models^119^. In a specialized three-dimensional (3D) model of a bone-like microenvironment, the direct addition of PGE2 to the cultures also caused the breast cancer cells to break dormancy and proliferate^120^.

## Potential application of lipid droplets in pre-metastatic niche detection and targeted therapy

The clinical establishment of PMN detection technology may help patients optimize the choice of monitoring and intervention during therapy. However, there is no effective clinical technique to detect PMNs. The observation that the PMN exhibits highly increased numbers of LDs and increased expression of LD-associated proteins/contents highlights the possibility of detecting LDs or LD-associated proteins/contents as biomarkers in PMNs. Recent advances in lipid detection, particularly in hyperspectral-stimulated Raman scattering microscopy, allow the assessment of the composition of LDs in a single cell^121^. Although it is still a long way from routine clinical use, this further raises the possibility of LDs as biomarkers in detecting PMNs. Indeed, an aberrant accumulation of cholesterol esterification in LDs was detected in high-grade and metastatic prostate cancers but not in benign lesions or normal tissue^122,123^. Considering that S100A8/A9 released from MDSCs reflects the establishment of an immunosuppressive environment in the lung PMN, researchers have developed a method that uses antibody-based SPECT to detect S100A8/A9 in vivo as an imaging marker for pre-metastatic tissue priming^74^. Because simply detecting the presence of LDs in PMNs lacks specificity, more LD-related molecules and detection systems should be tested.

Targeted therapies directed against the LD-dependent establishment of PMNs to stop metastasis may be a promising strategy for cancer therapeutics. As we have previously discussed, LDs modulate PMN formation as specialized intracellular sites for signal transduction. Although no specific LD inhibitors have been identified so far, different types of drugs and gene knockout of LD-associated proteins have been shown to inhibit LD formation. Treatment with the COX-2 inhibitor celecoxib significantly abolished tumor cell adhesion to endothelial cells and reduced the recruitment of BMDCs in pre-metastatic lungs^54^. Inhibition of LD formation by aspirin correlated with inhibition of both PGE2 production and tumor cell proliferation^22^. A similar effect was obtained with LD inhibition by C75, a drug that targets FA synthase^22^. Furthermore, targeting both the primary tumor and the PMN is an effective strategy to inhibit tumor metastasis. FASN inhibition with orlistat, which is an antiobesity drug, reduces both neovascularization and melanoma cell proliferation ex vivo^124^. Further studies also revealed that orlistat decreases the migration of LECs and enhances lymphatic permeability, thus reducing the size of LN metastasis^97^. The ubiquity of LDs underscores why the specific characteristics of LDs in PMNs need to be further explored to contribute to more precise diagnosis and treatment.

## Conclusions

Major advances in the understanding of LD regulation of cancer metastasis have been achieved in recent years. However, as an organelle that is centrally involved in cellular lipid balance and cellular signaling, our current understanding of LD dynamics, heterogeneity, and function in the PMN is still very limited. Throughout this review, we discussed features that involve LDs in PMN formation, pointing out recent evidence that associates these organelles with the six currently accepted characteristics of the PMN. This should allow researchers to integrate all LD-dependent PMN events into one comprehensive view. Ultimately, the ability to control LDs in the PMN will further improve the efficacy and specificity of cancer therapy.

Several questions remain regarding LD dynamics during PMN formation: 1. What is the specific relationship between LD biogenesis and lipid metabolism or lipid rafts in the PMN? 2. What are the spatiotemporal regulatory functions of LD characteristics at different stages of PMN development? 3. How can we evaluate the heterogeneity of LDs during the distinct phases of PMN development? 4. What are the differences in the effects of tumor-derived LDs and immune cell-derived or stromal cell-derived LDs on the PMN? 5. How do LDs mediate the crosstalk between tumor cells and stromal cells in the PMN? 6. What is the causal relationship between LD dynamic changes and PMN formation? 7. After the primary tumor is surgically removed, does the effect of the LDs on the PMN terminate? 8. LDs are organelles and cannot be knocked out because they are necessary for the normal function of the cell; therefore, how can the mechanism of LD formation be studied? 9. How can we evaluate the reliability and effectiveness of LDs as molecular markers of tumor metastasis? 10. Some results indicate that metastasis-initiating cells specifically rely on dietary lipids to promote metastasis^104^. Therefore, more work is needed to explore the effect of an HFD on PMN formation.

How to translate the basic research findings into clinical practices is another challenge. A better understanding of the cell biology of LDs and further exploration of the functional significance of LDs in the PMN will facilitate a breakthrough in cancer therapy and provide a rationale for clinical trials. This may be crucial for the application of novel therapies in the early stage of cancer.

## Methods

### Cell culture and quantification of neutral lipids

The THP-1 cells and siha cells were purchased from ATCC and cultured according to their guidelines in a humid atmosphere with 5% CO_2_. The cell lines were tested for authenticity in 2015 using short tandem repeat (STR) genotyping and screened for mycoplasma contamination (e-Myco Mycoplasma PCR Detection Kit; iNtRON). For potential PMN-associated macrophage on PMA-THP-1 monocytes, culture medium was replaced with 10% FBS DMEM supplemented with 5% human cancer cell serum (siha cell).The lipophilic fluorescence dye BODIPY 493/503 (Invitrogen) was used for monitoring the neutral lipid accumulation in cells. The actin antibody was used to depict the cytoskeleton.

### Analysis of gene expression and tumor-infiltrating immune cells

To investigate the correlation between the expression of PLIN2 and tumor-infiltrating immune cells (B cells, CD4+ T cells, CD8+ T cells, neutrophils, macrophages, and DCs), we applied the TIMER web server (https://cistrome.shinyapps.io/timer/), which is a comprehensive resource for systematical analysis of immune infiltrates across diverse cancer types available in the TCGA database.

## References

[1] Guan X (2015). Cancer metastases: challenges and opportunities. Acta Pharm. Sin. B.

[2] Valastyan S, Weinberg RA (2011). Tumor metastasis: molecular insights and evolving paradigms. Cell.

[3] Fidler IJ, Nicolson GL (1976). Organ selectivity for implantation survival and growth of B16 melanoma variant tumor lines. J. Natl Cancer Inst..

[4] Paget S (1989). The distribution of secondary growths in cancer of the breast. 1889. Cancer Metast. Rev..

[5] Paget S (1889). The distribution of secondary growths in cancer of the breast. Lancet.

[6] Hart IR, Fidler IJ (1980). Role of organ selectivity in the determination of metastatic patterns of B16 melanoma. Cancer Res..

[7] Welch DR, Hurst DR (2019). Defining the hallmarks of metastasis. Cancer Res..

[8] Liu Y, Cao X (2016). Characteristics and significance of the pre-metastatic niche. Cancer Cell.

[9] Chin AR, Wang SE (2016). Cancer tills the premetastatic field: mechanistic basis and clinical implications. Clin. Cancer Res..

[10] Kaplan RN (2005). VEGFR1-positive haematopoietic bone marrow progenitors initiate the pre-metastatic niche. Nature.

[11] Sleeman JP (2012). The metastatic niche and stromal progression. Cancer Metast. Rev..

[12] Hsu Y (2020). Bone-marrow-derived cell-released extracellular vesicle miR-92a regulates hepatic pre-metastatic niche in lung cancer. Oncogene.

[13] Guo Y (2019). Effects of exosomes on pre-metastatic niche formation in tumors. Mol. Cancer.

[14] Wang Y, Ding Y, Guo N, Wang S (2019). MDSCs: key criminals of tumor pre-metastatic niche formation. Front. Immunol..

[15] Giles AJ (2016). Activation of hematopoietic stem/progenitor cells promotes immunosuppression within the pre-metastatic niche. Cancer Res..

[16] Parton RG, Martin S (2006). Lipid droplets: a unified view of a dynamic organelle. Nat. Rev. Mol. Cell Biol..

[17] Cruz ALS, Barreto EDA, Fazolini NPB, Viola JPB, Bozza PT (2020). Lipid droplets: platforms with multiple functions in cancer hallmarks. Cell Death Dis..

[18] Thiam AR, Farese JRV, Walther TC (2013). The biophysics and cell biology of lipid droplets. Nat. Rev. Mol. Cell Biol..

[19] Itabe H, Yamaguchi T, Nimura S, Sasabe N (2017). Perilipins: a diversity of intracellular lipid droplet proteins. Lipids Health Dis..

[20] Aboumrad MH, Horn RJ, Fine G (1963). Lipid-secreting mammary carcinoma. Report of a case associated with Paget’s disease of the nipple. Cancer-Am. Cancer Soc..

[21] Wright DH (1968). Lipid content of malignant lymphomas. J. Clin. Pathol..

[22] Accioly MT (2008). Lipid bodies are reservoirs of cyclooxygenase-2 and sites of prostaglandin-E2 synthesis in colon cancer cells. Cancer Res..

[23] Bozza PT, Viola JPB (2010). Lipid droplets in inflammation and cancer. Prostaglandins Leukot. Essent. Fat. Acids (PLEFA).

[24] Olzmann JA, Carvalho P (2018). Dynamics and functions of lipid droplets. Nat. Rev..

[25] den Brok MH, Raaijmakers TK, Collado-Camps E, Adema GJ (2018). Lipid droplets as immune modulators in myeloid cells. Trends Immunol..

[26] Nath A, Chan C (2016). Genetic alterations in fatty acid transport and metabolism genes are associated with metastatic progression and poor prognosis of human cancers. Sci. Rep. -UK.

[27] Shang C (2018). LNMICC promotes nodal metastasis of cervical cancer by reprogramming fatty acid metabolism. Cancer Res..

[28] Long AP (2012). Lipid droplet de novo formation and fission are linked to the cell cycle in fission yeast. Traffic.

[29] Pol A, Gross SP, Parton RG (2014). Review: biogenesis of the multifunctional lipid droplet: lipids, proteins, and sites. J. Cell Biol..

[30] Wilfling F, Haas JT, Walther TC, Farese RV (2014). Lipid droplet biogenesis. Curr. Opin. Cell Biol..

[31] Robenek MJ (2004). Lipids partition caveolin‐1 from ER membranes into lipid droplets: updating the model of lipid droplet biogenesis. FASEB J..

[32] Murphy DJ, Vance J (1999). Mechanisms of lipid-body formation. Trends Biochem. Sci..

[33] Robenek H (2006). Adipophilin-enriched domains in the ER membrane are sites of lipid droplet biogenesis. J. Cell Sci..

[34] Wan HC, Melo RCN, Jin Z, Dvorak AM, Weller PF (2006). Roles and origins of leukocyte lipid bodies: proteomic and ultrastructural studies. FASEB J..

[35] Bozza PT, Magalhães KG, Weller PF (2009). Leukocyte lipid bodies—biogenesis and functions in inflammation. Biochim. Biophys. Acta (BBA) - Mol. Cell Biol. Lipids.

[36] Suzuki M, Shinohara Y, Ohsaki Y, Fujimoto T (2011). Lipid droplets: size matters. Microscopy.

[37] Yang H, Galea A, Sytnyk V, Crossley M (2012). Controlling the size of lipid droplets: lipid and protein factors. Curr. Opin. Cell Biol..

[38] Berndt N (2019). Characterization of lipid and lipid droplet metabolism in human HCC. Cells.

[39] Li FF (2018). Interaction with adipocytes induces lung adenocarcinoma A549 cell migration and tumor growth. Mol. Med. Rep..

[40] Hinson ER, Cresswell P (2009). The antiviral protein, viperin, localizes to lipid droplets via its N-terminal amphipathic alpha-helix. Proc. Natl Acad. Sci. USA.

[41] Prévost C (2018). Mechanism and determinants of amphipathic helix-containing protein targeting to lipid droplets. Dev. Cell..

[42] Gong J (2011). Fsp27 promotes lipid droplet growth by lipid exchange and transfer at lipid droplet contact sites. J. Cell Biol..

[43] Krahmer N (2011). Phosphatidylcholine synthesis for lipid droplet expansion is mediated by localized activation of CTP: phosphocholine cytidylyltransferase. Cell Metab..

[44] Dvorak AM (1983). Lipid bodies: cytoplasmic organelles important to arachidonate metabolism in macrophages and mast cells. J. Immunol..

[45] Weller PF, Ackerman SJ, Nicholson-Weller A, Dvorak AM (1989). Cytoplasmic lipid bodies of human neutrophilic leukocytes. Am. J. Pathol..

[46] Melo RCN, Fabrino DL, Dias FF, Parreira GG (2006). Lipid bodies: structural markers of inflammatory macrophages in innate immunity. Inflamm. Res..

[47] Bose D (2019). Inhibition of TGF-β induced lipid droplets switches M2 macrophages to M1 phenotype. Toxicol. Vitr..

[48] Umamaheswaran S, Dasari SK, Yang P, Lutgendorf SK, Sood AK (2018). Stress, inflammation, and eicosanoids: an emerging perspective. Cancer Metastasis Rev..

[49] Wang D, DuBois RN (2010). Eicosanoids and cancer. Nat. Rev. Cancer.

[50] Chen L (2017). cPLA2α mediates TGF-β-induced epithelial–mesenchymal transition in breast cancer through PI3k/Akt signaling. Cell Death Dis..

[51] Yu W (1998). Co-compartmentalization of MAP kinases and cytosolic phospholipase A2 at cytoplasmic arachidonate-rich lipid bodies. Am. J. Pathol..

[52] Wculek SK, Malanchi I (2015). Neutrophils support lung colonization of metastasis-initiating breast cancer cells. Nature.

[53] McLemore TL (1988). Profiles of prostaglandin biosynthesis in normal lung and tumor tissue from lung cancer patients. Cancer Res..

[54] LIU S (2014). Vascular endothelial growth factor plays a critical role in the formation of the pre-metastatic niche via prostaglandin E2. Oncol. Rep..

[55] Greenhough A (2009). The COX-2/PGE2 pathway: key roles in the hallmarks of cancer and adaptation to the tumour microenvironment. Carcinogenesis.

[56] Tunset HM, Feuerherm AJ, Selvik LM, Johansen B, Moestue SA (2019). Cytosolic phospholipase A2 alpha regulates TLR signaling and migration in metastatic 4T1 cells. Int. J. Mol. Sci..

[57] Melo RCN (2011). Lipid bodies in inflammatory cells: structure, function, and current imaging techniques. J. Histochem. Cytochem..

[58] Gupta GP (2007). Mediators of vascular remodelling co-opted for sequential steps in lung metastasis. Nature.

[59] Roulin K (1999). The fatty acid-binding heterocomplex FA-p34 formed by S100A8 and S100A9 is the major fatty acid carrier in neutrophils and translocates from the cytosol to the membrane upon stimulation. Exp. Cell Res..

[60] Watanabe A (2008). The S100A8-serum amyloid A3-TLR4 paracrine cascade establishes a pre-metastatic phase. Nat. Cell Biol..

[61] Tomita T, Sakurai Y, Ishibashi S, Maru Y (2011). Imbalance of Clara cell-mediated homeostatic inflammation is involved in lung metastasis. Oncogene.

[62] Kitamura T, Qian B, Pollard JW (2015). Immune cell promotion of metastasis. Nat. Rev. Immunol..

[63] Liu Y, Cao X (2016). Immunosuppressive cells in tumor immune escape and metastasis. J. Mol. Med..

[64] den Brok MH (2016). Saponin-based adjuvants induce cross-presentation in dendritic cells by intracellular lipid body formation. Nat. Commun..

[65] Herber DL (2010). Lipid accumulation and dendritic cell dysfunction in cancer. Nat. Med..

[66] Zhang Y (2014). Fatty acid-binding protein E-FABP restricts tumor growth by promoting IFN-beta responses in tumor-associated macrophages. Cancer Res..

[67] Wright HJ (2017). CDCP1 drives triple-negative breast cancer metastasis through reduction of lipid-droplet abundance and stimulation of fatty acid oxidation. Proc. Natl Acad. Sci. USA.

[68] Lucenay KS (2016). Cyclin E associates with the lipogenic enzyme ATP-citrate lyase to enable malignant growth of breast cancer cells. Cancer Res..

[69] Cotte AK (2018). Lysophosphatidylcholine acyltransferase 2-mediated lipid droplet production supports colorectal cancer chemoresistance. Nat. Commun..

[70] Kalinski P (2011). Regulation of immune responses by prostaglandin E2. J. Immunol..

[71] Hossain F (2015). Inhibition of fatty acid oxidation modulates immunosuppressive functions of myeloid-derived suppressor cells and enhances cancer therapies. Cancer Immunol. Res..

[72] Al-Khami AA (2017). Exogenous lipid uptake induces metabolic and functional reprogramming of tumor-associated myeloid-derived suppressor cells. OncoImmunology.

[73] Wu H (2017). Oleate but not stearate induces the regulatory phenotype of myeloid suppressor cells. Sci. Rep.-UK.

[74] Eisenblaetter M (2017). Visualization of tumor-immune interaction—target-specific imaging of S100A8/A9 reveals pre-metastatic niche establishment. Theranostics.

[75] He Y (2018). Transitory presence of myeloid-derived suppressor cells in neonates is critical for control of inflammation. Nat. Med..

[76] Prima V, Kaliberova LN, Kaliberov S, Curiel DT, Kusmartsev S (2017). COX2/mPGES1/PGE2 pathway regulates PD-L1 expression in tumor-associated macrophages and myeloid-derived suppressor cells. Proc. Natl Acad. Sci. USA.

[77] Goc J (2014). Dendritic cells in tumor-associated tertiary lymphoid structures signal a Th1 cytotoxic immune contexture and license the positive prognostic value of infiltrating CD8+ T cells. Cancer Res..

[78] Bougnères L (2009). A role for lipid bodies in the cross-presentation of phagocytosed antigens by MHC Class I in dendritic cells. Immunity.

[79] Stoth M (2019). Splenectomy reduces lung metastases and tumoral and metastatic niche inflammation. Int. J. Cancer.

[80] Ogawa F (2014). Prostanoid induces premetastatic niche in regional lymph nodes. J. Clin. Investig..

[81] Ramakrishnan R (2014). Oxidized lipids block antigen cross-presentation by dendritic cells in cancer. J. Immunol..

[82] Veglia F (2017). Lipid bodies containing oxidatively truncated lipids block antigen cross-presentation by dendritic cells in cancer. Nat. Commun..

[83] Chen XW (2017). CYP4A in tumor-associated macrophages promotes pre-metastatic niche formation and metastasis. Oncogene.

[84] Wu H (2019). Lipid droplet‐dependent fatty acid metabolism controls the immune suppressive phenotype of tumor‐associated macrophages. EMBO Mol. Med..

[85] Zhang Y (2014). Fatty acid-binding protein E-FABP restricts tumor growth by promoting IFN-responses in tumor-associated macrophages. Cancer Res..

[86] Niu Z (2017). Caspase-1 cleaves PPARγ for potentiating the pro-tumor action of TAMs. Nat. Commun..

[87] Li S (2015). Lipopolysaccharide induces inflammation and facilitates lung metastasis in a breast cancer model via the prostaglandin E2-EP2 pathway. Mol. Med. Rep..

[88] Seguin F (2012). The fatty acid synthase inhibitor orlistat reduces experimental metastases and angiogenesis in B16-F10 melanomas. Br. J. Cancer.

[89] Li Y (2014). Lipid droplets may lay a spacial foundation for vasculogenic mimicry formation in hepatocellular carcinoma. Med. Hypotheses.

[90] Kukla M (2010). Association between liver steatosis and angiogenesis in chronic hepatitis C. Pol. J. Pathol..

[91] Ju RJ (2014). Liposomes, modified with PTD (HIV-1) peptide, containing epirubicin and celecoxib, to target vasculogenic mimicry channels in invasive breast cancer. Biomaterials.

[92] Borin T, Angara K, Rashid M, Achyut B, Arbab A (2017). Arachidonic acid metabolite as a novel therapeutic target in breast cancer metastasis. Int. J. Mol. Sci..

[93] Huang Y (2009). Pulmonary vascular destabilization in the premetastatic phase facilitates lung metastasis. Cancer Res..

[94] Bovay E (2018). Multiple roles of lymphatic vessels in peripheral lymph node development. J. Exp. Med..

[95] Ye Y, Liu S, Wu C, Sun Z (2015). TGFβ modulates inflammatory cytokines and growth factors to create premetastatic microenvironment and stimulate lung metastasis. J. Mol. Histol..

[96] Wong BW (2017). The role of fatty acid β-oxidation in lymphangiogenesis. Nature.

[97] Bastos DC (2017). Effects of fatty acid synthase inhibitors on lymphatic vessels: an in vitro and in vivo study in a melanoma model. Lab. Investig..

[98] Lala PK, Nandi P, Majumder M (2018). Roles of prostaglandins in tumor-associated lymphangiogenesis with special reference to breast cancer. Cancer Metast. Rev..

[99] Nandi P (2017). PGE2 promotes breast cancer-associated lymphangiogenesis by activation of EP4 receptor on lymphatic endothelial cells. BMC Cancer.

[100] Xin X (2012). Targeting COX-2 and EP4 to control tumor growth, angiogenesis, lymphangiogenesis and metastasis to the lungs and lymph nodes in a breast cancer model. Lab. Investig..

[101] Boschi F, Rizzatti V, Zamboni M, Sbarbati A (2015). Models of lipid droplets growth and fission in adipocyte cells. Exp. Cell Res..

[102] Jung JI (2015). High-fat diet-induced obesity increases lymphangiogenesis and lymph node metastasis in the B16F10 melanoma allograft model: roles of adipocytes and M2-macrophages. Int. J. Cancer.

[103] Gao Y (2019). Metastasis organotropism: redefining the congenial soil. Dev. Cell.

[104] Pascual G (2016). Targeting metastasis-initiating cells through the fatty acid receptor CD36. Nature.

[105] Ladanyi A (2018). Adipocyte-induced CD36 expression drives ovarian cancer progression and metastasis. Oncogene.

[106] Chkourko Gusky H, Diedrich J, MacDougald OA, Podgorski I (2016). Omentum and bone marrow: how adipocyte-rich organs create tumour microenvironments conducive for metastatic progression. Obes. Rev..

[107] Suetsugu A (2015). Imaging the interaction of pancreatic cancer and stellate cells in the tumor microenvironment during metastasis. Anticancer Res..

[108] Thomas D, Radhakrishnan P (2020). Pancreatic stellate cells: the key orchestrator of the pancreatic tumor microenvironment. Adv. Exp. Med. Biol..

[109] Hata T (2017). Fatty acid–mediated stromal reprogramming of pancreatic stellate cells induces inflammation and fibrosis that fuels pancreatic cancer. Pancreas.

[110] Mikuriya Y (2015). Fatty liver creates a pro-metastatic microenvironment for hepatocellular carcinoma through activation of hepatic stellate cells. Int. J. Cancer.

[111] Shah T, Wildes F, Kakkad S, Artemov D, Bhujwalla ZM (2016). Lymphatic endothelial cells actively regulate prostate cancer cell invasion. NMR Biomed..

[112] Deep G (2020). Exosomes secreted by prostate cancer cells under hypoxia promote matrix metalloproteinases activity at pre-metastatic niches. Mol. Carcinog..

[113] Costa-Silva B (2015). Pancreatic cancer exosomes initiate pre-metastatic niche formation in the liver. Nat. Cell Biol..

[114] Corbet C (2020). TGFβ2-induced formation of lipid droplets supports acidosis-driven EMT and the metastatic spreading of cancer cells. Nat. Commun..

[115] Thews O, Riemann A (2019). Tumor pH and metastasis: a malignant process beyond hypoxia. Cancer Metast. Rev..

[116] Hoshino A (2015). Tumour exosome integrins determine organotropic metastasis. Nature.

[117] Li J (2018). An omega-3 polyunsaturated fatty acid derivative, 18-HEPE, protects against CXCR4-associated melanoma metastasis. Carcinogenesis.

[118] Pan M, Hou M, Chang H, Hung W (2008). Cyclooxygenase-2 up-regulates CCR7 via EP2/EP4 receptor signaling pathways to enhance lymphatic invasion of breast cancer cells. J. Biol. Chem..

[119] Panigrahy D (2012). Epoxyeicosanoids stimulate multiorgan metastasis and tumor dormancy escape in mice. J. Clin. Investig..

[120] Sosnoski DM, Norgard RJ, Grove CD, Foster SJ, Mastro AM (2015). Dormancy and growth of metastatic breast cancer cells in a bone-like microenvironment. Clin. Exp. Metast..

[121] Zhang C, Li J, Lan L, Cheng J (2017). Quantification of lipid metabolism in living cells through the dynamics of lipid droplets measured by stimulated raman scattering imaging. Anal. Chem..

[122] Li J (2016). Abrogating cholesterol esterification suppresses growth and metastasis of pancreatic cancer. Oncogene.

[123] Lee HJ (2018). Cholesterol esterification inhibition suppresses prostate cancer metastasis by impairing the Wnt/β-catenin pathway. Mol. Cancer Res..

[124] Carvalho MA (2008). Fatty acid synthase inhibition with Orlistat promotes apoptosis and reduces cell growth and lymph node metastasis in a mouse melanoma model. Int. J. Cancer.

